# Shotgun proteomics datasets acquired on *Gammarus pulex* animals sampled from the wild

**DOI:** 10.1016/j.dib.2019.104650

**Published:** 2019-10-12

**Authors:** Duarte Gouveia, Yannick Cogne, Jean-Charles Gaillard, Christine Almunia, Olivier Pible, Adeline François, Davide Degli-Esposti, Olivier Geffard, Arnaud Chaumot, Jean Armengaud

**Affiliations:** a Laboratoire Innovations technologiques pour la Détection et le Diagnostic (Li2D), Service de Pharmacologie et Immunoanalyse (SPI), CEA, INRA, F-30207 Bagnols-sur-Cèze, France; b Irstea, UR RiverLy, Laboratoire d’écotoxicologie, centre de Lyon-Villeurbanne, F-69625 Villeurbanne, France

**Keywords:** Amphipods, Cadmium contamination, Ecotoxicology, Intrapopulation variability, Proteomics, Sentinel animal

## Abstract

This data article associated with the manuscript “Comparative proteomics in the wild: accounting for intrapopulation variability improves describing proteome response in a Gammarus pulex field population exposed to cadmium” refers to the shotgun proteomics analysis performed on 40 *Gammarus pulex* animals sampled from the wild. Proteins were extracted, digested with trypsin, and the resulting peptides were identified by tandem mass spectrometry. Here, we present the list of proteins from males and the list of proteins from females that are differentially detected between the Brameloup and the Pollon populations. Data are available via ProteomeXchange with identifiers PXD013656 and PXD013712, respectively.

**Table:** Specifications Table

Subject area	*Biology*
More specific subject area	*Proteomics, Tandem mass spectrometry*
Type of data	*Table, figure*
How data was acquired	*Mass spectrometry, data-dependent analysis mode using a Thermo Q-Exactive HF instrument*
Data format	*Raw, filtered, analyzed.*
Experimental factors	*Global proteomes from animals sampled either at Brameloup or Pollon sites*
Experimental features	*Tandem mass spectrometry coupled to label-free quantification*
Data source location	*Bagnols-sur-Cèze, France* *Sampling at Brameloup river: 45°07′51″N; 4°25′00″E* *Sampling at Pollon river: 45°57′21″N; 5°15′44″E*
Data accessibility	*Data are with this article and the MS/MS raw files have been deposited to the ProteomeXchange Consortium via the PRIDE repository with the data identifier identifiers PXD013656 and 10.6019/PXD013656 for the G. pulex males, and PXD013712 and 10.6019/PXD013712 for the G. pulex females.*

**Table:** 

**Value of the Data** • These shotgun proteomic data are useful as they represent the first acquired on individual Gammarids sampled from the wild. • This dataset provided the core proteome of *Gammarus pulex* male and female animals. • Ecotoxicologists can directly benefit from these data as Gammarids are sentinel animals. • The data presented here can be used for studies of protein sequence variability between animals of the same population. • These data may be used for improving the annotation of gammarid genomes and transcriptomes and certifying protein sequences.

## 1 Data

**Fig. 1:**
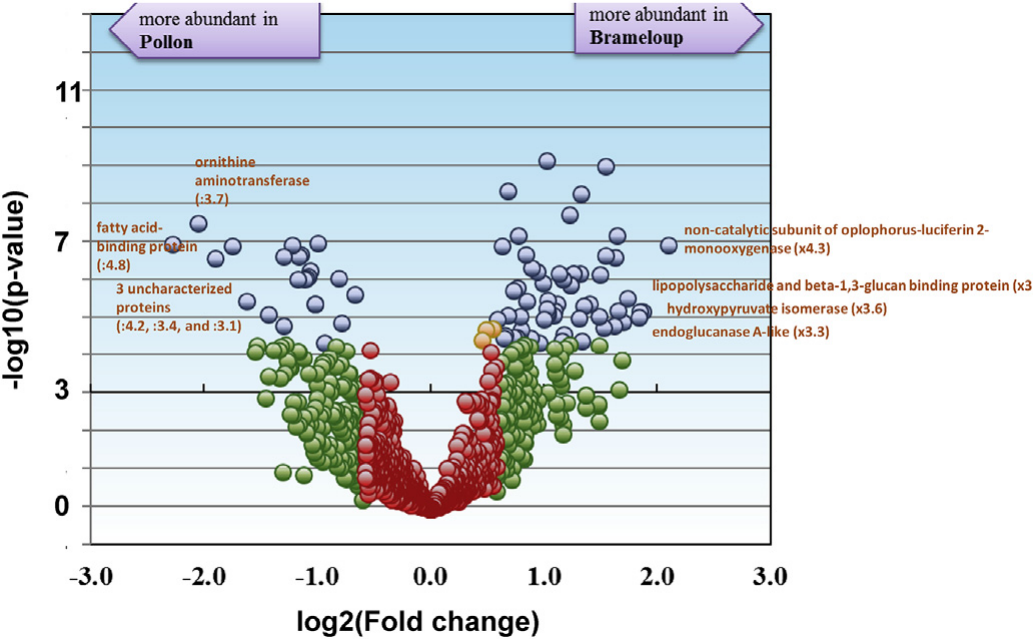
**Volcano plot showing the proteomics comparison of Brameloup versus Pollon male populations.** In blue are proteins satisfying both, the fold (≥1.5) and statistical criteria (Bonferroni corrected p value ≤ 0.05). In orange are the identifications that did not meet the fold criterion but have low p-values. In green are proteins satisfying the fold criterion but, most likely, this happened by chance. In red are proteins that did not meet the fold and p-value criteria (n = 10 per population).

**Fig. 2:**
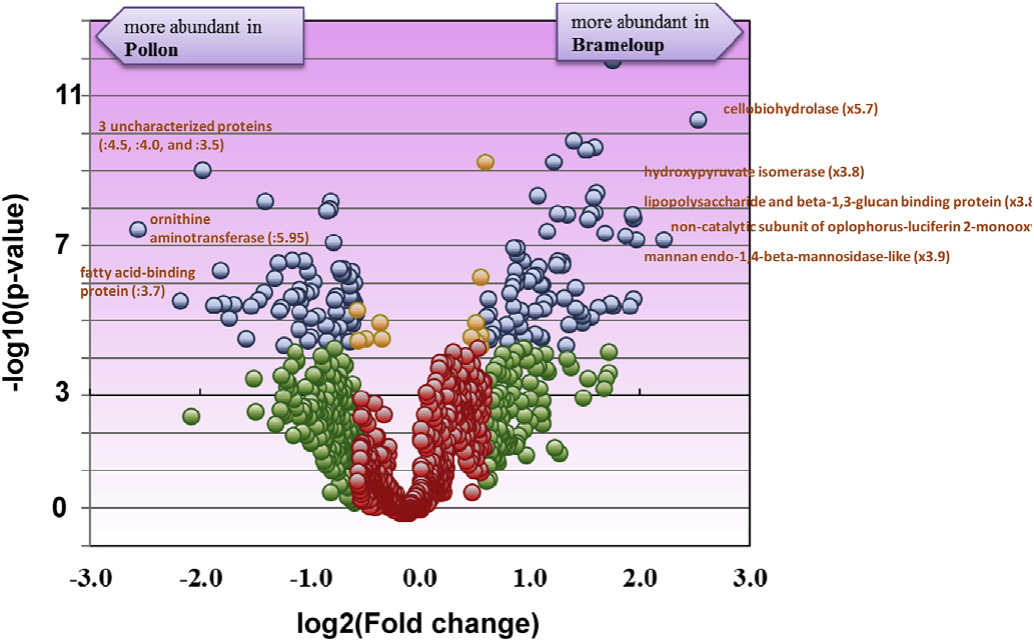
**Volcano plot showing the proteomics comparison of Brameloup versus Pollon female populations.** In blue are proteins satisfying both, the fold (≥1.5) and statistical criteria (Bonferroni corrected p value ≤ 0.05). In orange are the identifications that did not meet the fold criterion but have low p-values. In green are proteins satisfying the fold criterion but, most likely, this happened by chance. In red are proteins that did not meet the fold and p-value criteria (n = 10 per population).

The dataset presented in this article was generated through a proteogenomic study on two natural populations of the non-genome sequenced crustacean *Gammarus pulex* in the context of a larger project aimed at defining and validating molecular biomarkers by proteogenomics and targeted proteomics rely on a better knowledge of these sentinel animals [1]. The dataset allows studying the intrapopulation proteome variability of long-term exposed individuals (Brameloup population) in relation to non-contaminated individuals (Pollon population) as described in Ref. [2]. Table 1 shows the total number of peptide-spectrum matches, peptides, non-ambiguous peptides, and proteins obtained for the male and female datasets. Here, the core-proteome (a concept previously defined in Ref. [3]), *i.e.* the proteins detected systematically in all organisms from the same sex, accounts for 42 and 44% of the total proteome in the female and male dataset, respectively. Population-specific proteins (detected exclusively in organisms from either Pollon or Brameloup) account for 10% and 8% of the male and female proteomes. Supplementary Table S1 and Supplementary Table S2 give the list of identified proteins, their spectral counts, functional annotations, and the inter-population differential proteomic analysis for male and female organisms, respectively. The most relevant proteins with significant abundance changes for males and females are represented in Fig. 1 and Fig. 2, respectively. The two most overdetected proteins in the Brameloup population compared to the Pollon population are a monooxygenase (fold change x4.2) and a lipopolysaccharide and beta-1,3-glucan binding protein (x3.9) in males, and a cellobiohydrolase (x5.7) and the same monooxygenase (x4.6) in females. The two less detected proteins are a fatty acid-binding protein (−4.8x) and an uncharacterized protein (−4.15x) for the males, and the ornithine aminotransferase (−5.9x) and an uncharacterized protein (−4.5x) for females. Generally, the most differentially detected proteins report for similar functions in males and females. These proteins are implicated in oxidative stress, digestion, and host defense against microorganisms, which is in accordance with the fact that the animals come from distinct populations with different food sources and contamination levels. As discussed in Ref. [2], the continuous exposure to Cd in the Brameloup site may have induced a chronic Cd-based oxidative stress in these animals. Data were deposited in the PRIDE database and are available in ProteomeXchange under the identifiers PXD013656 and PXD013712, respectively.

**Table 1:** Statistics of the shotgun proteomics data acquired on *G. pulex* individuals.

	Individuals (n)	Peptide-spectrum matches	Peptide sequences	Non-ambiguous peptide sequences	Proteins	Core proteome
Male dataset	20	397465	11435	8134	1385	605
Female dataset	20	448228	12080	8658	1444	607

## 2 Experimental design, materials and methods

### 2.1 Animal sampling and protein extraction

Gammarids were collected from two distinct populations in the Pollon (45°57′21″N; 5°15′44″E) and Brameloup (45°07′51″N; 4°25′00″E) rivers, both located in mid-eastern France. Organisms were sorted using a 2–2.5 mm sieve in order to select only adults with similar size and age. Proteins were extracted from the whole body of each individual. Ten sexually mature males and females from each population (a total of forty animals) were weighed and transferred into 2 mL Screw Cap microtubes (Sarstedt), and rapidly frozen in liquid nitrogen. Proteins were extracted from the whole body of individual organisms. For this, each individual was homogenized through bead-beating in LDS buffer (20 μL per mg of organism) containing 26.5 mM Tris/HCl, 35.25 mM Tris base, 0.5% lithium dodecyl sulfate, 2.5% glycerol, 0.13 mM EDTA, 0.06 mM SERVA Blue G-250, and 0.04 mM phenol red, buffered at pH 8.5, and supplemented with 5% beta-mercaptoethanol. A 3.2 mm steel bead was added to each tube. Samples were submitted to 3 cycles of 30 sec of bead-beating (30 sec of pause between each cycle) at 7800 rpm in a Precellys Evolution instrument (Bertin Technologies). Samples were centrifuged at 10,000 *g* for 3 min, and the supernatant collected into a new tube. Protein extracts were incubated for 5 min at 99 °C, and subjected to a short electrophoretic migration (SDS-PAGE) in conditions similar as previously described [4]. Briefly, 20 μL of each protein extract was loaded into a well of a 4–12% polyacrylamide gel, and subjected to denaturing migration using a fixed voltage of 200V for 4 min. Proteins in the gel were stained with Coomassie SimplyBlue SafeStain (ThermoFisher Scientific) for 30 min, and then destained with milliQ water overnight. The whole-protein content from each well was then extracted as a single polyacrylamide band. The resulting forty individual bands were placed in a 96-well plate. Gel bands were further destained and dehydrated using methanol and acetonitrile. After drying in a speed vac, reduction of disulfide bonds was performed using 25 mM dithiothreitol (DTT) in 50 mM NH_4_HCO_3_ at 56 °C for 10 min, followed by alkylation of sulfhydryl groups with 55 mM iodoacetamide in 50 mM NH_4_HCO_3_ for 10 min, at room temperature and in the dark. Proteolysis of proteins was performed at 50 °C for 60 min with trypsin gold (Promega) in 50 mM NH_4_HCO_3_ supplemented with the ProteaseMAX (Promega) surfactant. Peptide solutions were acidified with trifluoroacetic acid (final concentration 0.5%) and transferred to mass spectrometry-compatible vials.

### 2.2 NanoLC-MS/MS analysis, identification and label-free quantitation of the animal proteomes

The peptide mixtures were analysed through data-dependent acquisition with a Q-Exactive HF tandem mass spectrometer (Thermo) including a high field orbitrap analyser and coupled to an UltiMate 3000 LC system (Dionex-LC Packings) as previously described [5]. Peptides were first desalted on a reverse-phase PepMap100 C18 μ-precolumn (5 μm, 100 Å, 300 μm i.d. × 5 mm, ThermoFisher), and then resolved onto a nanoscale C18 PepMap 100 capillary column (3 μm, 100 Å, 75 μm i.d. × 50 cm, ThermoFisher) with a 90-min gradient of CH3CN, 0.1% formic acid (solvent B), at a flow rate of 0.2 μL/min. The gradient used was: 4–25% of solvent B for 75 min, followed by 25–40% of solvent B for 15 min. Full scan mass spectra were acquired from *m/z* 350 to 1800 with an Automatic Gain Control (AGC) target set at 3 × 10^6^ ions and a resolution of 60,000. Within each scan cycle, the top 20 precursor ions were subjected to fragmentation through high-energy collisional dissociation. MS/MS scan was initiated when the AGC target reached 10^5^ ions with a threshold intensity of 17,000 and potential charge states of 2^+^ and 3^+^ after ion selection performed with a dynamic exclusion of 10 sec.

Raw MS/MS files were converted to.mgf files using Proteome Discoverer v1.4 (Thermo), and these were searched against a gender-specific RNAseq-derived database obtained previously by *de novo* assembly of *G. pulex* individual transcriptomes and identification of the most probable open reading frames [6]. The production of these transcriptomes and the construction of the protein databases are thoroughly described in Ref. [6]. Briefly, RNA-seq was performed on a single genotyped animal, the reads being assembled by a *de novo* strategy using Trinity v2.4 [7], and the resulting contigs being translated with Transdecoder v3.0.1 [7]. The *G. pulex* male database contains 111,751 putative protein sequences totalling 17,598,514 amino acids while the female database comprises 121,147 putative protein sequences totalling 22,904,430 amino acids. The search algorithm used for peptide assignment was Mascot Daemon version 2.3.2 (Matrix Science). MS/MS spectra interpretation was performed using the following parameters: full-trypsin specificity, maximum of one missed cleavage, mass tolerances of 5 ppm on the parent ion and 0.02 Da on the MS/MS, carboxyamidomethylated cysteine (+57.0215) as a fixed modification, and oxidised methionine (+15.9949) and deamidation of asparagine and glutamine (+0.9848) as variable modifications. All peptide-to-spectrum matches presenting a MASCOT peptide score with a p-value lower than 0.055, corresponding to a FDR of 2% as evaluated with the DecoyPyrat procedure [8], were filtered and assigned to polypeptide sequences. Only proteins with at least two different peptide sequences were considered for statistical comparison. Peptide-to-spectrum matches were counted for each polypeptide without applying the parsimony rule to avoid any quantitative bias between isoforms.

### 2.3 Statistical analysis

For comparing the two *G. pulex* populations, only peptide sequences detected at least once in each population were considered for each sex. All the identified proteins were label-free quantified. For each protein, spectral counts of these common peptides were normalized as initially proposed [9], *i.e.* one spectral count (+1) was added to each protein in each condition. Fold changes for protein abundances were evaluated using the Tfold method [10] and statistically validated with a Student test considering sample with unequal variance and bilateral repartition of abundances of proteins. Bonferroni correction was applied on p-values using the number of proteins detected with at least two different peptides for each sex. When comparing Brameloup and Pollon populations, proteins with a Bonferroni corrected p-value below 0.05 and a Tfold absolute value higher than 1.5 were considered as differentially expressed.

### 2.4 Data availability

The mass spectrometry and proteomics data have been deposited to the ProteomeXchange Consortium via the PRIDE [11] partner repository with the dataset identifiers PXD013656 and 10.6019/PXD013656 for the proteomes from *G. pulex* males, and PXD013712 and 10.6019/PXD013712 for the proteoms from *G. pulex* females.
